# Evaluating Antimicrobial Susceptibility Testing Methods for Cefiderocol: A Review and Expert Opinion on Current Practices and Future Directions

**DOI:** 10.3390/antibiotics14080760

**Published:** 2025-07-28

**Authors:** Stefania Stefani, Fabio Arena, Luigi Principe, Stefano Stracquadanio, Chiara Vismara, Gian Maria Rossolini

**Affiliations:** 1 Department of Biomedical and Biotechnological Sciences, University of Catania, 95128 Catania, Italy; s.stracquadanio@unict.it; 2 Department of Clinical and Experimental Medicine, University of Foggia, 71100 Foggia, Italy; fabio.arena@unifg.it; 3 UOC Microbiologia e Virologia, Azienda Ospedaliera Bianchi-Melacrino-Morelli di Reggio Calabria, 89121 Reggio Calabria, Italy; luigi.principe@gmail.com; 4 S.C. Microbiologia Clinica ASST Grande Ospedale Metropolitano Niguarda, 20162 Milan, Italy; chiara.vismara@ospedaleniguarda.it; 5 Department of Experimental and Clinical, University of Florence, 50100 Florence, Italy; gianmaria.rossolini@unifi.it; 6 Microbiology and Virology Unit, Careggi University Hospital, 50100 Florence, Italy

**Keywords:** cefiderocol, antimicrobial susceptibility testing, clinical microbiology

## Abstract

Background: Cefiderocol (FDC) presents challenges in antimicrobial susceptibility testing (AST). The reference standard is the broth microdilution (BMD) method with iron-depleted cation-adjusted Mueller-Hinton broth (ID-CAMHB). Still, it is cumbersome for routine clinical laboratory use, while variable accuracy has been reported with available commercial systems. Variability in interpretive criteria and areas of technical uncertainty (ATUs) further complicate assessments. Methods: This review and expert opinion presents: (1) an overview of non-susceptibility to FDC and then delves into the performance of current FDC AST methods for Enterobacterales, *Pseudomonas aeruginosa*, and *Acinetobacter baumannii* complex; (2) a practical decision framework to guide clinical microbiologists in making informed choices. Results and Conclusions: For Enterobacterales, including carbapenem-resistant Enterobacterales (CRE), and *Pseudomonas aeruginosa*, we propose disk diffusion (DD) as a preliminary screening tool to classify isolates as susceptible (S) or resistant (R). Confirmatory testing using the UMIC^®^ FDC system or the ID-CAMHB BMD method is recommended for R isolates. In cases of discrepancy, repeating the test with ID-CAMHB BMD is advised. Additionally, isolates falling within the ATU during DD testing should be retested using the UMIC^®^ system or ID-CAMHB BMD. For *A. baumannii* complex, since EUCAST breakpoints have not been defined yet, we propose a stepwise framework based on the first DD result: isolates with inhibition zones < 17 mm are considered non-susceptible and should be confirmed with standard BMD. Those between 17 and 22 mm require retesting with a commercial BMD method, with further confirmation recommended if S isolates with zones ≥ 23 mm may be considered S without additional testing.

## 1. Introduction

Antimicrobial resistance (AMR) among MDR Gram-negative (GN) bacteria represents a major global health concern, particularly in hospital and critical care settings. In Intensive Care Units (ICUs), MDR-GNs—primarily carbapenem-resistant Enterobacterales, *Pseudomonas aeruginosa*, and *Acinetobacter baumannii*—account for over one-third of nosocomial infections. These pathogens complicate treatment decisions, increase mortality, and contribute to substantial healthcare costs [1,2].

Recent antimicrobial resistance surveillance data in Europe collected by the Central Asian and European Surveillance of Antimicrobial Resistance (CAESAR) network and the European Antimicrobial Resistance Surveillance Network (EARS-Net), revealed that in this time frame third-generation cephalosporin resistance in *Klebsiella pneumoniae* was widespread in the WHO European Region, with 42% of the countries, especially in the southern and eastern parts, reporting high resistance levels of 50% or above [1]. Conversely, only 16% of the countries had resistance levels below 10%. According to the ECDC, carbapenem resistance in *K. pneumoniae* is substantially more common than in *Escherichia coli*: 33% of reporting countries documented resistance rates ≥ 25% in *K. pneumoniae*, and 18% reported rates ≥ 50%, while resistance in *E. coli* remains rare and generally below 1%. Resistance to carbapenems in *P. aeruginosa* and *Acinetobacter* spp. also showed significant geographic variability [1,2]. Specifically, 14% of countries reported high levels of carbapenem resistance in *P. aeruginosa* (50% or more), while in *Acinetobacter* spp. 56% of the countries reported 50% or above resistance levels, predominantly in the southern and eastern parts of Europe [1].

Despite the introduction of new β-lactamase inhibitor combinations, the demand for effective antibacterial drugs continues to increase. In this context, the introduction in 2019 of cefiderocol (FDC, Shionogi & Co., Ltd., Osaka, Japan) a siderophore cephalosporin, is a pivotal advancement in the treatment of MDR-GN infections. FDC is approved for a broad spectrum of serious infections caused by GN pathogens, including strains resistant to available treatments, as it is relatively stable against hydrolysis by many serine- and metallo- β -lactamases (MBLs) [3,4,5,6].

The dual penetration mechanism of FDC, through passive diffusion and active transport via the iron transport system, promotes effective delivery and achievement of high concentrations within the bacterial periplasm. This allows FDC to bypass common resistance mechanisms such as porin loss in carbapenemase-resistant (CR) strains, a feature that may confer an advantage over other β-lactams like ceftolozane or meropenem. Like other β-lactams, FDC binds penicillin-binding proteins, disrupts cell wall synthesis, and induces bacterial cell death [3,4,7]. Given its siderophore-mediated cell entry mechanism, accurate susceptibility testing for cefiderocol requires iron-depleted conditions to replicate its in vivo activity and ensure reliable MIC determination.

Implementing and using standardized and reproducible methods for antibiotic susceptibility testing (AST) for FDC is crucial for its clinical application, patient safety, and surveillance. However, it remains a complex and challenging endeavor [8]. This review and expert opinion aims to offer an overview of global prevalence and general mechanisms of non-susceptibility to FDC and evaluate the performance of current AST practices, proposing decision frameworks for FDC AST with Enterobacterales, *P. aeruginosa*, and *A. baumannii* complex, which can be directly accessed in the Expert Opinion and Discussion section.

## 2. Materials and Methods

First, a comprehensive state-of-the-art review was conducted on the mechanisms of on-susceptibility and the types of AST methodologies.

A focused search was then conducted in PubMed using the keywords “antimicrobial susceptibility testing,” “cefiderocol susceptibility testing,” “Cefiderocol,” and “Gram-negative bacteria” to center on studies evaluating AST performance. These terms were combined using Boolean operators, such as AND and OR, to cover the spectrum of AST methodologies and non-susceptibility mechanisms associated with FDC.

Studies were included in the final evaluation if:

- Focused on Enterobacterales, *P. aeruginosa*, and *A. baumannii* complex clinical isolates.
- Reported on comparing AST methodologies specifically for FDC versus broth microdilution method (BMD).

Studies were excluded if not written in English or if they were editorials, reviews, or meta-analyses. Our search strategy identified 53 papers on PubMed, of which 13 were selected for our final review.

All authors independently screened all titles, abstracts, and content to select eligible studies. Discrepancies at any stage were discussed and resolved unanimously. The final decision frameworks reflect a shared consensus, and no major dissenting views were expressed.

### 2.1. Data Synthesis Approach

Data from the selected studies were qualitatively synthesized, summarizing findings related to the performance (in terms of Categorical Agreement [CA], Essential Agreement [EA], Very Major Errors [VME], Major Errors [ME], minor errors [mE], and bias parameters). This synthesis aimed to highlight trends, consensus points among studies, and notable discrepancies in results or methodologies.

### 2.2. Collection of Expert Opinions

Expert opinions were gathered through a blend of face-to-face and virtual meetings (three in total) orchestrated by the coordinator, Stefania Stefani. These meetings involved key microbiology experts delving into their practical experiences, challenges faced, and evaluations on AST for FDC.

### 2.3. Terminology Notes

Throughout the manuscript, the following definitions apply:

○ Resistance refers to classification based on clinical breakpoints (EUCAST, CLSI, FDA).
○ Reduced susceptibility is used in the absence of species-specific clinical breakpoints, typically in reference to MICs above PK/PD thresholds.
○ Non-susceptibility is retained when referring to prevalence/mechanism data or published surveillance reports that used this term.

## 3. Non-Susceptibility to FDC: Global Prevalence and General Mechanisms

According to recent surveillance data, non-susceptibility prevalence to FDC (NS-FDC) remains low overall [2,9]. A recent systematic review and meta-analysis encompassing 82,035 clinical isolates (period 2020–2023) revealed that the prevalence of NS-FDC, according to EUCAST breakpoints, exhibited variability across different bacterial species. For instance, rates among Enterobacterales, *P. aeruginosa*, and *A. baumannii* were 3.0% (95%CI 1.5–6.0%), 1.4% (95%CI 0.5–4.0%), and 8.8% (95%CI 4.9–15.2%), respectively. Higher rates were found in carbapenem-resistant NDM-producing Enterobacterales with a prevalence of 38.8% (95%CI 22.6–58.0%) [9].

Four potential factors may contribute to the emergence of NS-FDC:

- Expression of metallo-β-lactamase NDM and/or β-lactamases (e.g., SHV, PER, and VEB) [10,11,12,13,14,15,16,17,18,19]. In *A. baumannii*, combining FDC with avibactam has been shown to reduce MIC, indicating that β -lactamase activity in this species contributes to reduced susceptibility [20]. In addition, increased copy numbers of the bla_NDM-5_ gene due to translocation events have enhanced NDM production, leading to decreased activity of FDC. However, the extent to which bla_NDM-5_ expression contributes to FDC reduced susceptibility remains to be fully elucidated [21].
- Structural alterations in β-lactamases, such as AmpC, for example, the region encoding the R2 loop [22,23,24]. Furthermore, comparable variations in Oxacillinases include specific point mutations in the Ω loop of OXA-2 (Ala149Pro and Asp150Gly) and the OXA-10 subgroup (Trp154Cys and Gly157Asp). Additionally, the OXA-10 subgroup exhibits a duplication of Thr206 and Gly207 in the β5–β6 loop [25]. In vivo and in vitro emergence of NS-FDC due to KPC variants (e.g., KPC-41 and KPC-50) has also been reported [19].
- Mutations in FDC’s PBP3 target (i.e., 4-amino acid insertion at position 333) reduce FDC’s access to the specific transpeptidase pocket [26,27,28]. Mutations like these do not directly confer non-susceptibility but are commonly observed in isolates that produce β-lactamases, such as NDM-type. Such mutations can contribute to reaching a certain level of clinical non-susceptibility when associated with other mechanisms [26,27,28].
- Mutations or reduced expression of genes involved in iron transport pathways, particularly siderophore receptor genes and inner membrane proteins (e.g., pirA, cirA, ton, piuA, fecA, kfu, TonB, ExbB, and ExbD) [21,29,30,31,32,33,34,35,36,37,38].

In addition, emerging mechanisms such as CpxS-mediated regulation of iron uptake have been implicated in FDC non-susceptibility, particularly in *P. aeruginosa*. Such adaptations have been observed in both cooperative protection via siderophores and the selection of heteroresistant subpopulations under prolonged FDC therapy. These findings underscore the need to consider enzymatic and structural determinants, regulatory pathways, and population dynamics when evaluating FDC activity [37,38].

Reduced susceptibility to FDC development during treatment has been observed mainly through translocation events that result in multiple copies of *bla_NDM_* genes in Enterobacterales [39]. High-level resistance may also arise in NDM producers due to the selection of mutants in the iron transport systems [40]. Clinical isolates may exhibit various mechanisms [41,42]. Further, *P. aeruginosa* clinical isolates prone to ceftolozane-tazobactam and ceftazidime-avibactam resistance overall have high cefiderocol MICs, suggesting a role of the pseudomonal-derived cephalosporinase and MexAB systems in cefiderocol non-susceptibility [43]. Notably, for all these Gram-negative classes, literature emphasizes that FDC susceptibility loss is generally via multiple mechanisms compounded rather than one [33,44].

In addition, recent studies have identified clinical carbapenem-resistant Enterobacterales isolates with increased MICs of FDC (MIC > 4 mg/L) that have never been exposed to the drug [45]. This may be expected, given that FDC targets the same PBP3 as other cephalosporins and monobactams. Additionally, variants in the omega loop of β-lactamases can develop following exposure to other β-lactams.

These mechanisms observed in patients without prior FDC exposure and numerous reports of low-barrier non-susceptibility emergence to cefiderocol—both in vitro and in clinical settings—underscore the urgent need to refine AST methodologies and better inform clinical treatment decisions.

## 4. Current Standards and AST Methodologies for FDC

Broth Microdilution (BMD) is the gold standard for assessing FDC susceptibility. This method necessitates using an Iron–Depleted–Cation–Adjusted Mueller-Hinton Broth (ID-CAMHB) with a final iron concentration of ≤0.03 μg/mL, requiring lengthy preparation and posing challenges for routine implementation [46]. However, concerns have been raised regarding variability in BMD results even when using ID-CAMHB prepared according to standards. Differences among commercial reagents, such as manufacturer-supplied versus pure reference cefiderocol powder, may impact MIC values, sometimes skewing results toward higher in vitro activity [47].

Furthermore, the interpretation of the BMD method is complex due to the requirement, specific to the European Committee on Antimicrobial Susceptibility Testing (EUCAST), to observe the first well where a reduction (not absence) of growth occurs, indicated by a button size of less than 1 mm in diameter or the presence of a light haze/turbidity. Interpreting BMD results for FDC can be even more challenging with certain microorganisms, such as *Acinetobacter* spp., owing to potential trailing effects [8,48] characterized by weak growth across multiple wells [49,50,51].

Several commercial tests have been developed to determine the MIC of FDC using BMD, each offering specific, tailored features. However, some of these methods have raised concerns regarding accuracy and reproducibility. In August 2022, EUCAST highlighted the difficulty in interpreting results from specific commercial FDC tests due to these limitations [50]. Similarly, the CLSI Antimicrobial Susceptibility Testing Subcommittee emphasized the need for careful interpretation and standardization of testing procedures [47].

Sensititre™ EUMDROXF by Thermo Fisher, a semi-automated system, uses a standard culture medium rather than an iron-depleted medium. It has been withdrawn from the market due to concerns over its reliability. The ComASP^®^ cefiderocol microdilution panel (Liofilchem, Roseto degli Abruzzi, Italy) and UMIC^®^ cefiderocol strips (Bruker Daltonics GmbH & Co. KG, Bremen, Germany) are manual systems that employ a “ready-to-use” iron-depleted culture medium and are currently among the few available options complying with guideline requirements. Given the variability among BMD-based commercial systems and limited access to standardized testing, EUCAST and CLSI recommend initiating FDC testing with the disk diffusion (DD) method as a first-line approach. If performed adequately with an FDC 30 µg disk according to EUCAST standard recommendations for non-fastidious organisms and calibrated with quality control strains, DD has been considered predictive of susceptibility and resistance outside areas of technical uncertainty (ATUs) [48]. Most studies to date have used the previous ATU definitions; the impact of the updated values remains to be fully evaluated. While narrowing ATUs may improve testing accuracy, some data suggest it could also increase the risk of false negatives [46,52].

Thus, the DD method, currently endorsed by EUCAST as a frontline approach, offers a practical alternative by circumventing the need for an iron-depleted medium [53]. This is because the iron in the standard Mueller-Hinton (MH) broth is believed to form complexes with the agar matrix, simulating an iron-depleted environment. It has been demonstrated that iron-regulated outer membrane proteins (iROMPs), which function as receptors for siderophore-iron complexes, are expressed on the surface of cells from colonies grown on agar plates. On the contrary, these proteins are absent from the cell membranes of bacteria cultured in standard CAMHB [54]. However, DD carries the burden of ATUs, where strains overlap with MICs on either side of FDC’s susceptibility/resistance breakpoint. This overlap can lead to ambiguity in the interpretation of results, thus presenting a challenge for clinicians attempting to make informed treatment decisions [8]. EUCAST advised ignoring the ATU until alternative validated methods were developed to resolve interpretive uncertainties using the zone diameter breakpoints in the breakpoint table [53]. EUCAST also highlighted that the results depend on the quality of the materials used in all AST methods and antimicrobial agents. However, EUCAST did not systematically investigate all products, disks, or MH media [53]. In the evaluation of FDC 30 µg disks from Liofilchem, Mast, and Oxoid alongside MH agars from BBL, bioMérieux, Bio-Rad, Liofilchem, and Oxoid, it was found that Oxoid disks and Bio-Rad MH agar produced zone diameters exceeding the acceptable limits for both quality control strains and clinical samples. Combining Oxoid disks with Bio-Rad MH agar further exacerbated this issue, leading to larger zone diameters [53]. CA, EA, and bias parameters were evaluated according to the ISO 20776-2:2021 guidelines for BMD. EUCAST breakpoints served as standards. Acceptable levels were set at 90% for CA and EA, with a permissible bias range of ±30% [53].

There is controversy over appropriate ATUs and narrowing setpoints; some suggest changes will improve accuracy, while some cite risks of higher false negative rates. Importantly, discrepancies exist among CLSI guidelines (ed. 34, 2024), EUCAST (v.14, 2024), and FDA (version updated on 12 November 2024) as shown in Table 1, suggesting that even with DD AST, clinical microbiologists lack consensus on approaches to detecting FDC resistance. Further, EUCAST has yet to release BMD and DD breakpoints for two gram-negatives: *Acinetobacter* spp. and *Stenotrophomonas maltophilia* [50,55]. EUCAST provides an essential guide for interpreting inhibition zones, suggesting that diameters larger than 17 and 20 mm for disks containing 30 mcg FDC typically correspond to MIC values BELOW the PK/PD susceptibility breakpoint (<2 mg/L) for *A. baumannii* and *S. maltophilia*, respectively [55].

Regarding AST methods that rely on alternative approaches, different from BMD and DD, a recent study aimed to develop a rapid culture-based test (namely Rapid Cefiderocol NP test) to detect FDC resistance in MDR Enterobacterales [56]. This test leverages the metabolic activity of glucose in the presence of FDC at a concentration of 64 mg/L in an iron-depleted CAMHB medium. It also uses a color change from red to yellow in red phenol, a pH indicator, to visually signify bacterial growth [56]. Evaluated using 74 clinical enterobacterial isolates, the test demonstrated a high sensitivity of 98% and a specificity of 91% compared to the standard BMD method [55]. Remarkably, the Rapid Cefiderocol NP test delivered results within three hours, significantly reducing the time required for susceptibility testing [56]. Additional confirmation of this test across various laboratories, involving a broader and more varied collection of FDC-non-susceptible and -susceptible enterobacterial isolates, is essential to validate its efficacy and reliability comprehensively. An equivalent Rapid Cefiderocol NP test for *A. baumannii* has been developed [57]. This test relies on bacterial viable cells reducing resazurin to resorufin, which shifts the color from blue to violet or pink, indicating bacterial growth in the presence of FDC at a concentration of 38.4 mg/L. Using 95 randomly selected *A. baumannii* isolates, the test demonstrated a sensitivity of 95.5% and a specificity of 100% compared to BMD, with only a single VME identified [57]. These findings suggest that NP-based methods may represent a rapid alternative for screening purposes. However, these tests are not currently commercially available, and further comparative studies—including functional in vitro assays and clinical correlation analyses—are required to establish their reliability, practical utility, and potential advantages over existing gold-standard methods.

ATU limitations and discrepancies of breakpoints prompt questions about the best ways to assess FDC AST. The Gradient Strip Test (specifically the MIC Strip Test by Liofilchem) (Roseto degli Abruzzi, Italy) uses a strip with a gradient of FDC concentrations to determine MIC. Still, it is only validated for *P. aeruginosa* and should not be used with other pathogens (Enterobacterales or *A. baumannii*) as it may lead to reporting of false susceptibles (VME) [58,59,60]. Agar dilution was assessed by Albano et al., who used 610 g-negative bacilli. While tested against standard and iron-depleted broth microdilution (BMD), the agar dilution method showed poor agreement with BMD, variable categorical agreement (47–93%), and frequent errors. It tended to yield higher MICs and failed to meet accuracy thresholds, indicating it is unreliable for FDC MIC testing [61].

However, it could be essential to underline that while ID-BMD remains the reference method, limitations have been reported, particularly in non-fermenters such as *A. baumannii*, where reproducibility issues and incomplete alignment with clinical outcomes have been described elsewhere [62]. Ibrahim et al. developed the SuperFDC medium, which is not commercially available, to detect Enterobacterales, *P. aeruginosa*, and *A. baumannii* strains with FDC reduced susceptibility. The medium, which consisted of 8 mg/L FDC added to iron-deficient agar, was tested on 68 susceptible and 33 resistant GN bacteria with various β-lactam resistance mechanisms. The SuperFDC demonstrated 97% sensitivity and 100% specificity, with only 3% VME compared to the reference BMD. Furthermore, it performed satisfactorily, with a 10–103 CFU/mL detection limit in spiked stool tests [63].

## 5. Variability in Testing Outcomes Across Different Microorganisms

This section synthesizes AST outcomes only on Enterobacterales, *A. baumannii*, and *P. aeruginosa*, as these are the primary GN bacteria responsible for difficult-to-treat infections. The MIC and inhibition zone diameters of these bacteria have been reported to fall below and above the susceptibility breakpoints, sometimes within ATU [46,64]. On the other hand, species such as *Burkholderia pseudomallei*, *Achromobacter* spp., and *S. maltophilia* are usually found to be susceptible to FDC, displaying low MIC90 values and broad inhibition zones, which facilitate a more straightforward interpretation of the data [46,64,65].

While results are reported per microorganism due to methodological and breakpoint-specific differences, we highlight key patterns in agreement, error rates, and interpretive challenges across studies to support comparative insight.

### 5.1. Enterobacterales

**Figure 1:**
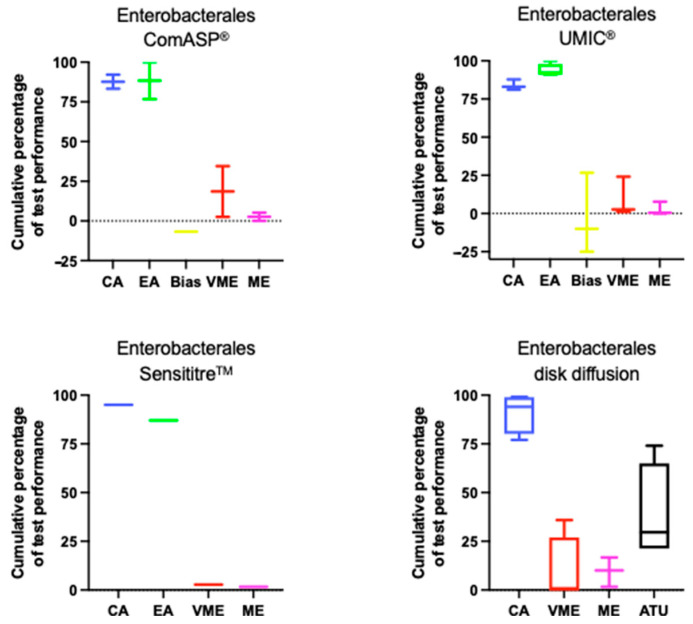
Performance of cefiderocol AST for Enterobacterales. Boxplots illustrate the distribution of categorical agreement (CA), essential agreement (EA), bias, very major errors (VME), major errors (ME), and where present area of technical uncertainty (ATU) across ComASP^®^, UMIC^®^, Sensititre™, and DD. Data are aggregated from studies included in this review.

Figure 1 shows the distribution of key performance metrics for ComASP^®^, UMIC^®^, Sensistitre^TM^ and DD among Enterobacterales, based on data from studies included in this review. Full details are available in Supplementary Materials (S1).

Three out of eight studies utilized the standard homemade BMD method as their reference, whereas the others employed commercial BMD or frozen panels.

Matuschek et al. evaluated the reproducibility of FDC MIC determination using ID-CAMHB BMD (frozen 96-well microtitre plates—Thermo Scientific, Oakwood, GA, USA) and EUCAST DD across 263 isolates, including *E. coli* and *K. pneumoniae*. Although not included in S1due to the absence of CA or EA data, the study tested 30 μg FDC disks from two manufacturers (Liofilchem and Mastdisc) on cation unsupplemented MHA plates prepared in-house from two manufacturers (Oxoid, Thermo Fisher Scientific, Basingstoke, UK) and BBL (Becton Dickinson, Sparks, NV, USA). DD showed overall reproducibility and highlighted a zone overlap (18–22 mm) between susceptible (≤2 mg/L) and resistant (>2 mg/L) isolates, underscoring the relevance of confirmatory MIC testing within the ATU range. The study also noted high MIC variability in wild-type strains, potentially linked to differences in iron transporter and siderophore expression [66].

Accordingly, other authors have concluded that while BMD remains the reference standard for AST in Enterobacterales, DD can provide a reliable alternative for initial susceptibility testing [67]. Morris et al. used BMD and DD with research-use-only 30 μg cefiderocol Mastdisc (Mast Group Ltd., Bootle, UK) to evaluate the susceptibility in 58 isolates of carbapenem-resistant Enterobacterales (CRE) and 50 isolates of non-fermentative GN bacilli [67]. Results indicated a susceptibility rate ranging from 72% (FDA criteria) to 90% (CLSI criteria), a variation that underscores the impact of different regulatory standards on susceptibility outcomes. With DD interpreted according to the EUCAST criteria, a non-susceptibility of 65% and 66% was found compared to 74% and 72% for the FDA criteria and 87% and 89% for CLSI, respectively, with HardyDisks and Mastdiscs. CA ranged from 75% to 90%, with ME present in percentages varying from 8% to 25%, mE from 0 to 19%, and VME from 0 to 20%, considering the variability of disks and breakpoints evaluated [67]. Bonnin et al. focused specifically on CRE, applying various methodologies including CE-IVD BMD plate (ThermoFisher) using regular MH broth, MTS (Liofilchem), and DD (Liofilchem), which were compared to a frozen BMD plate prepared with iron-depleted MH broth [60]. Interestingly, the study’s first phase assessed comparability between commercial CE-IVD BMD (Sensititre™ EUMDROXF) and standard BMD.

The method showed strong concordance with the reference BMD (CA 95%, EA 87%, VME 2.8%, ME 1.6%), while MTS strips performed poorly, with a VME rate of 94.9%, highlighting their limited reliability in this setting [60]. However, test strips are not approved for Enterobacterales; they are only for *P. aeruginosa*. The second phase of the study was a prospective comparison between DD and CE-IVD BMD with EUCAST breakpoints among 827 Enterobacterales (76.7% carbapenemase producers, including 426 OXA-48-like, 55 VIM-, 118 NDM-, and 9 KPC-producers) [60]. The results revealed that 77% of the isolates were accurately categorized by DD, although with a considerable rate of VMEs (35.9%) and a MEs of 1.6%. Notably, of the VME cases, 21 out of 22 had MIC values of 4 mg/L, suggesting an acceptable edge of technical error within two dilutions. The study revealed broad MIC distributions and notable variability in inhibition zones, particularly among carbapenemase-producing or highly resistant strains. Approximately 21.3% of isolates fell within the ATU, requiring BMD confirmation. Overall, the method showed performance comparable to standard BMD for assessing FDC susceptibility [60]. Focusing on a set of 60 CRE, another study by Emeraud et al. concentrated on the performance of two commercial tests, ComASP^®^ FDC microdilution panel and UMIC^®^ FDC, compared with a frozen panel containing FDC in ID-CAMHB prepared by Thermo Fisher (Sensititre™ CML1FEUD plate) and stored according to EN ISO 20776-1:2019 and CLSI document M07, which was used as the reference method on 60 CREs [68]. The study revealed an FDC sensitivity rate of 70% when tested with ComASP^®^ FDC microdilution panel, with an EA of 76.7% and a CA of 83%. However, the method also showed a high VME rate of 34.5% (10 strains falsely classified as susceptible), indicating discrepancies in resistance detection compared with the BMD standard. In contrast, UMIC^®^ FDC aligned better with the BMD results, with a sensitivity rate of 58.3%, an EA of 91.7%, and a CA of 83.3%. The VME rates were 24.1% (7 strains falsely classified as susceptible) and 6.9% of the VMEs out of the EA, respectively [68]. The studies by Bianco et al. in 2023 and 2024 have also yielded significant findings on the AST for FDC in Enterobacterales and other GN bacteria [69,70]. The 2023 study by Bianco et al. FDC testing was performed using DD according to EUCAST guidelines. Cation-adjusted MHA (Becton-Dickinson, Franklin Lakes, NJ, USA) and a FDC 30 µg disk (Oxoid Ltd., Basingstoke, UK) were used. Sequentially, the ComASP^®^ FDC microdilution panel and the reference BMD were used in parallel to test FDC on isolates that presented ATU or non-interpretable results by DD [69].

The ComASP^®^ panel showed high concordance with BMD (CA 92.1%) in isolates with uninterpretable DD results, with only three discrepancies—one being a VME—all near the MIC threshold. Among CREs within the ATU, 20% were resistant by both methods [69]. The DD method allowed the assessment of FDC susceptibility in 78.6% of Enterobacterales. Furthermore, 23.7% of strains exhibiting ATU results were resistant according to the reference BMD method, with MICs ranging from 4 to 16 mg/L. Additionally, 20.5% of CRE showing ATUs were determined to be resistant by the ComASP^®^ FDC microdilution panel and the reference BMD technique [69].

In 2024, Bianco et al. evaluated UMIC^®^ FDC and DD to assess FDC susceptibility across a broader range of GN bacteria, including 90 Enterobacterales isolates [70]. The UMIC^®^ FDC demonstrated an EA of 81.1%, CA of 91.1%, and a bias of +26.7%. It showed only one VME where the MIC was 2 mg/L compared to the reference 4 mg/L, and seven MEs, primarily with MIC 4 mg/L versus the reference 2 mg/L. A general trend of MIC overestimation was noted (bias + 24.2%), leading to 12 MEs (7.4%). Most VME and ME fell within the EA range (+2-fold dilution). For strains with a reference MIC below 0.5 mg/L, over 80% of MICs were outside the EA [70]. DD exhibited a CA of 81.1% and an EA of 91.1%. High rates of ATU were observed in the Enterobacterales (37.8%). Only 11.8% of cases falling into the ATU corresponded to MICs in the resistance range [70]. In 2023, a study by Bovo et al. specifically examined the performance of FDC ASTs on 75 clinical isolates of KPC-producing *K. pneumoniae* [71]. The authors utilized three testing methods: a standard BMD fresh panel, DD, and MTS. DD showed a CA of 92% and a ME of 16.7%. There were no VMEs, indicating that DD is a valuable method for the initial screening [71]. MTS had a CA of 90.7% and showed a higher rate of VME at 17.9%, indicating discrepancies in measuring the resistance. The agreement was even lower at 82.7% between DD and MTS, with ME significantly higher at 30.2%, reflecting the inconsistencies between these two methods [71]. Finally, another recent study, presented at ESCMID 2024 by Leonildi and colleagues, explored the susceptibility of 30 NDM-producing *K. pneumoniae* isolates to FDC by comparing the ComASP^®^ FDC microdilution panel and UMIC^®^ FDC, alongside DD, to the BMD reference method. DD showed inhibition zones ranging from 20–24 mm, which in 74% of cases fell into the ATU, complicating the interpretation. The study found 100% EA and CA between BMD and commercial tests [52]. Key findings are summarized in Box 1.

Box 1 **Summary of key findings for Enterobacterales.**

DimensionKey pointsAgreement Average CA: ComASP^®^ 87.7%; UMIC^®^ 84%; Sensititre™ 95%; DD 91.05%
Average EA: ComASP^®^ 88.35%; UMIC^®^ 93.6%; Sensititre™ 87%ErrorsBias range: ComASP^®^ −6.7%; UMIC^®^ −25—+26.7%; Sensititre™ ND

Average VME: ComASP^®^ 18.5%; UMIC^®^ 9.3%; Sensititre™ 2.8%; DD 9%
Average ME: ComASP^®^ 2.6%; UMIC^®^ 2.7%; Sensititre™ 1.6%; DD 9.4%
Average DD ATU: 38.6%Test ConditionsDisk/media combinations highly influence DD performance.
Bio-Rad MH agar and Oxoid disks may produce inflated zone diameters. 
20–30% of isolates fall within ATU, requiring confirmatory testing.

### 5.2. Pseudomonas aeruginosa

**Figure 2:**
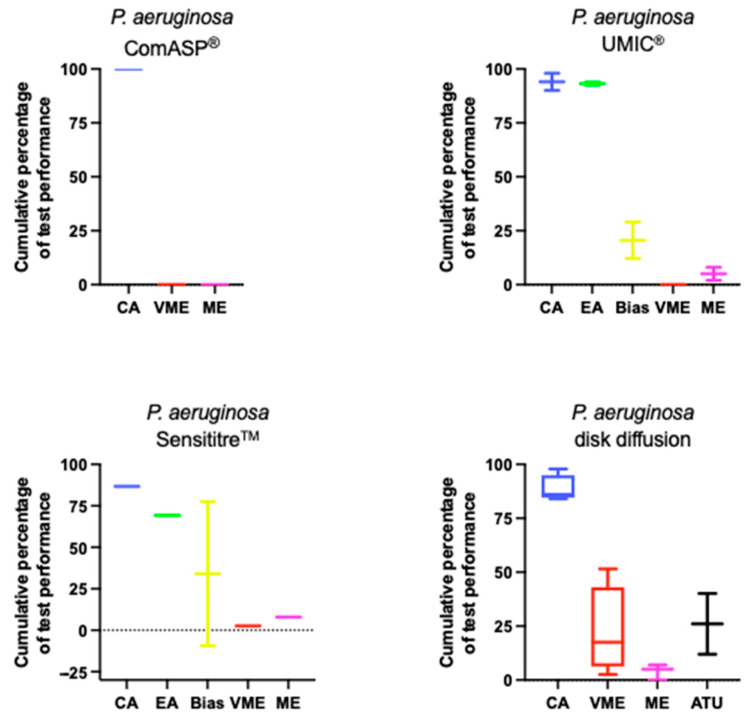
Performance of cefiderocol AST for *P. aeruginosa*. Boxplots illustrate the distribution of categorical agreement (CA), essential agreement (EA), bias, very major errors (VME), major errors (ME), and where present area of technical uncertainty (ATU) across ComASP^®^, UMIC^®^, Sensititre™, and DD. Data are aggregated from studies included in this review.

Figure 2 reports the overall performance of various AST methods for *P. aeruginosa*. Full details are available in Supplementary Materials (S2). Two of the five studies utilized the standard homemade BMD method as their reference, while the others employed commercial BMD or frozen panels.

Morris et al. reported wide variability in FDC susceptibility rates for carbapenem-resistant *P. aeruginosa* (57–93%), depending on the breakpoint criteria used, with CLSI breakpoints yielding the highest susceptibility [67]. Correlation between DD (HardyDisks and Mastdiscs) and BMD showed variability: 4 of 9 isolates appeared more susceptible and 5 more resistant by DD, unlike Enterobacterales, which tended to show higher susceptibility by DD. Both DD methods demonstrated acceptable CA, ranging from 79%. (HardyDisks with EUCAST breakpoints) 93% (HardyDisks and Mastdiscs with CLSI breakpoints) [67]. Dortet et al. evaluated the performance of UMIC^®^ FDC against standard BMD in various GN bacteria, including *P. aeruginosa*, in terms of analytical (inter-operator, execution cycle, site, execution day, batch) and clinical performance. The overall reproducibility of the method was high (>95%) for all parameters, and the overall clinical performance according to the ISO 20776-2.2021 target with EA >90% and −30% < bias < 30%. In particular, the study found that UMIC^®^ FDC provided reliable susceptibility testing results, showing a CA of 98.0% (95% CI: 89.3–99.6%) and EA of 93.9% (95% CI: 83.5–97.9%) for *P. aeruginosa*. The test performed better in non-fermenters than in Enterobacterales, where it tended to underestimate MICs—likely due to the higher prevalence of MBL producers, which were associated with elevated FDC MICs [72]. In Bianco et al. 2024, the UMIC^®^ FDC method aligned closely with BMD, although, as already mentioned, the effectiveness varied between different approaches and bacterial strains [70]. It is worth underlining that before carrying out the evaluations on 13 FDC-susceptible non-fermenting GNB (MIC ≤ 2 mg/L), Bianco et al. 2024 carried out in vitro resistance induction experiments to have a greater breadth of MICs for testing [70]. With this step, 48 isolates with different degrees of reduced susceptibility or frank resistance to FDC were obtained and included for the evaluations of both methods. CA among *P. aeruginosa* strains was 90% (n = 36/40) with an EA rate of 92.5% (n = 37/40). In this study, DD generally showed a high CA ranging from 94.9 to 100% regardless of the disk manufacturer in Enterobacterales, *P. aeruginosa*, and *A. baumannii*, according to EUCAST breakpoints. One VME was observed in *P. aeruginosa* for Oxoid (23 mm versus reference MIC, 16 mg/L). *P. aeruginosa* isolates with zone diameters falling within the ATU range of 14–22 mm demonstrated a higher resistance rate (68.7%) than Enterobacterales [70]. The ATU limit for *P. aeruginosa* was lowered to 20–21 mm in the EUCAST v14 update, which would result in several cases being excluded. In contrast, the ATU defined by CLSI and FDA (14–22 mm) has not been modified. These discrepancies likely stem from differences in dataset size, clinical correlations, and timing of breakpoint updates. Further harmonization and prospective validation are needed to refine DD interpretive criteria across systems. In Bianco et al. 2023, 11.9% of *P. aeruginosa* isolates exhibited inhibition zones inside the ATU. All *P. aeruginosa* tested susceptible (MICs range: 0.06–2 mg/L) with ComASP^®^ cefiderocol microdilution panel and reference BMD [69]. Disk diffusion allowed the determination of FDC susceptibility in 88.1% of strains, with all VIM-producing *P. aeruginosa* showing MIC values in the susceptible range (2 mg/L) [69]. Devoos et al. examined a collection of 150 clinical strains of *P. aeruginosa* resistant to ceftazidime (MIC > 8 mg/L), imipenem (MIC > 4 mg/L), and ceftolozane/tazobactam (MIC > 4 mg/L). 73 FDC susceptibility was tested using DD (three disk brands on six MHAs), MTS, and Sensititre microplates, compared to reference BMD. Only DD achieved an acceptable overall CA. Sensititre overestimated MICs (CA 86.7%, EA 69.3%, bias +68.2%), while MTS underestimated them (same CA and EA, bias −30.4%), correctly identifying just 50% of resistant strains [73]. Finally, the overall rate of CA for the 18 combinations of disks and MHA applied to the collection of clinical strains was 84.0%, with variations ranging from 78.0% to 89.3%. Acceptable CA rates, irrespective of the disk brand (excluding ATU), were achieved using Becton Dickinson (ranging from 94.0% to 95.5%), bioMerieux (ranging from 91.8% to 93.0%), and Mast MHA (ranging from 91.1% to 92.7%) [73]. It is worth noting that 20 isolates displayed microcolonies in the zone of inhibition for at least one combination of disks and MHA. These were more frequently observed with the Mast disc and MHA from Becton Dickinson, BioMérieux, Mast, and Oxoid [73]. While DD proved acceptable for assessing FDC susceptibility, substantial variability in inhibition zones was observed across disk–MHA combinations, including with the quality control strain, likely due to batch-dependent differences in MHA iron content (0.35–1.03 mg/L) [73]. Key findings are summarized in Box 2.

Box 2 **Summary of key findings for *P. aeruginosa*.**

DimensionKey PointsAgreementAverage CA: ComASP^®^ 100%; UMIC^®^ 94%; Sensititre™ 86.7%; DD 88.5%
Average EA: ComASP^®^ ND%; UMIC^®^ 93.2%; Sensititre™ 69.3%ErrorsBias range: ComASP^®^ ND; UMIC^®^ +12.12—+29%; Sensititre™ −9.3—+77.5 
Average VME: ComASP^®^ 0%; UMIC^®^ 0%; Sensititre™ 2.7%; DD 22.25%
Average ME: ComASP^®^ 0%; UMIC^®^ 5%; Sensititre™ 8%; DD 4%
Average DD ATU: 25.9%
Few VMEs reported, mainly in isolates with borderline MICs. 
MEs increase when MICs are slightly overestimated (e.g., 4 mg/L vs. 2 mg/L).Test ConditionsZone diameter variability is affected by the manufacturer of agar and the disk. 
ATU inhibition zones (20–22 mm) may be associated with reduced susceptibility.

### 5.3. Acinetobacter baumannii

**Figure 3:**
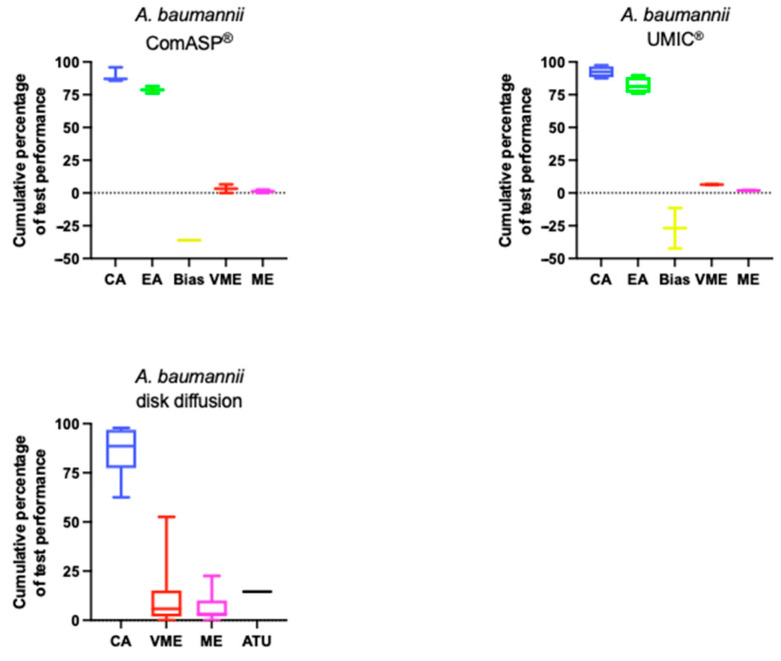
Performance of cefiderocol AST for *A. baumannii*. Boxplots illustrate the distribution of categorical agreement (CA), essential agreement (EA), bias, very major errors (VME), major errors (ME), and where present area of technical uncertainty (ATU) across ComASP^®^, UMIC^®^, and DD. Data are aggregated from studies included in this review.

Studies comparing the performance of various AST methods to a reference BMD method for *A. baumannii* are detailed in Figure 3. Full details are available in Supplementary Materials (S3). Four of seven studies used the standard homemade BMD method as their reference, while the others used commercial BMD or frozen panels.

It is important to note that for *Acinetobacter* spp., although EUCAST (2024) has not set specific FDC clinical breakpoints, there is currently only a PK/PD breakpoint (≤2 mg/L or ≤17 mm) defined. Meanwhile, CLSI provides breakpoints for A. baumannii complex (≤4 mg/L or ≥15 mm as susceptible and ≥16 mg/L as resistant) [47,52]. Therefore, we refer to isolates with MICs exceeding the PK/PD breakpoint as “reduced susceptibility”; however, this does not mean clinical resistance and should be interpreted cautiously.

Jeannot et al. tested the standard BMD and DD with three types of disks (Mast Diagnostic, Liofilchem, and Oxoid), UMIC^®^ FDC, and ComASP^®^ cefiderocol microdilution panel among 97 clinical isolates of *A. baumannii* with reduced susceptibility to at least one carbapenem. DD assays were carried out following the EUCAST guidelines. Using the reference BMD method, 43.3% of the strains were classified as strains with a reduced susceptibility to FDC [58]. For the comparison between BMD and the ComASP^®^ cefiderocol microdilution panel, EA was recorded at 81.4% and CA at 95.9%, with biases of −36.1% and +28.9%, respectively. In the BMD and UMIC^®^ FDC matchup, EA was 78.4%, CA was 93.8%, and bias was −42.3%. Four strains were sensitive to one of the two commercial BMD methods but not susceptible to the reference BMD; two were misclassified by both [58].

In the comparison between BMD and MTS, EA was reported at 59.8%, with a CA of 76.3%. The bias was −48.4%; however, the lower value observed was −62.9%. Using gradient strips, 55% of strains with a reduced susceptibility to FDC were incorrectly classified as susceptible [58]. When comparing BMD with DD, CA varied between 72.2% and 81.4%, depending on the type of disk used. The study identified microcolonies within the inhibition zones on all three types of disks, affecting 10 isolates (eight non-susceptible and two susceptible). While susceptible strains were correctly identified, resistant strains were misclassified in 42.9% (MAST), 50% (Liofilchem), and 64.3% (Oxoid) of cases. These errors were unrelated to β-lactam resistance mechanisms or strain backgrounds but correlated with gene expression; notably, PER-type β-lactamase and NDM producers were frequently resistant [58]. In the same study, the Authors also suggest that DD inhibition zones between 17 and 22 mm may include isolates that are non-susceptible by BMD and propose a higher cut-off of 22 mm to reduce the risk of underestimation of reduced susceptibility [58]. EUCAST has recently received these suggestions and updated its guidelines accordingly, although official breakpoints have yet to be defined [74].

Similarly, Kolesnik-Goldmann and colleagues utilized a collection of 100 carbapenem-resistant *v* strains to compare the performance of BMD with fresh panels, DD on two commercial ID-CAMH agar plates (from bioMérieux and Liofilchem), and a homemade version, UMIC^®^ FDC and ComASP^®^ cefiderocol microdilution panel. Additionally, MTS was performed on two commercial CAMH agar plates (BioMerieux and Liofilchem) and a homemade ID-CAMH [59]. The study used both the CLSI clinical breakpoints and the EUCAST PK-PD breakpoint, and the methods were extended with whole-genome sequencing and typing for thorough evaluation [59]. In specific comparisons, BMD against the ComASP^®^ cefiderocol microdilution panel yielded an EA of 76% and CA rates of 86% according to CLSI and 88% according to EUCAST standards, respectively, with VME reported at 6/100 with CLSI and 7/100 with EUCAST readings. UMIC^®^ FDC showed 76% EA and CA values of 86% (CLSI) and 89% (EUCAST), with VMEs slightly higher under EUCAST (9%) than CLSI (3%), indicating variability across interpretive standards [59]. The MTS demonstrated variability influenced by the type of media used. Using the homemade ID-CAMH, the EA was the highest at 75%, while with commercial media from Biomerieux and Liofilchem, it dropped to 57% and 44%, respectively. The CA remained similar across all media types at 87%, 85%, and 88%. However, VME varied more substantially, recorded at 2%, 10%, and 6.9%, respectively, highlighting media choice’s significant impact on the accuracy of susceptibility tests [59]. Morris et al. reported a CA of 64% for both CLSI and EUCAST when comparing DD to Sensititre™ BMD, with VMEs of 0% (CLSI) and 33% (EUCAST), and MEs of 20% and 25%, respectively [67].

The study by Bianco et al. 2023 showed a high CA of 97.9% with no VMEs, highlighting a highly reliable agreement between DD and the standard BMD reference method. Only a 2.1% occurrence of MEs suggests a strong performance in accurately categorizing strains [69]. MIC determinations obtained by ComASP^®^ cefiderocol microdilution panel and reference BMD on the 7 A. baumannii isolates that achieved ATU by DD revealed that 6 isolates (85.7%) were NS (MICs range: 4–16 mg/L) [65].

Bianco and colleagues also detected high agreement rates in comparing DD and BMD in 2024, who reported a CA of 94.9% and a CA of 89.7% in comparing DD or UMIC^®^ FDC with BMD, respectively. Also in this study, susceptibility was interpreted according to EUCAST clinical breakpoints (v. 13.0 2023). The study also noted MEs and VMEs at 3% for DD versus BMD, reflecting minor discrepancies that might affect interpretation but generally suggest a reliable testing method [70]. Dortet et al. reported 90% EA and 84.1% CA for UMIC^®^ FDC, with a bias of −11.4% and an ME rate of 6.8%. Results were interpreted using non-species-specific PK/PD EUCAST breakpoints (susceptible ≤ 2 mg/L) [72].

Finally, Liu et al. compared DD with standard BMD using CLSI and EUCAST breakpoints. Overall, CA was high: 98.1% (CLSI) and 97.0% (EUCAST), with VME rates of 0.9% and 1.9%, respectively. For carbapenem-susceptible *A. baumannii*, CA was 100% with no errors. In carbapenem-resistant strains, CA remained high (97.5% CLSI; 96.2% EUCAST), with low VMEs. Among difficult-to-treat isolates, CA was 97.6% (CLSI) and 95.7% (EUCAST), with VMEs of 1.2% and 3.1% [75]. Key findings are summarized in Box 3.

Box 3 **Summary of key findings for *A. baumannii*.**

DimensionKey pointsAgreementAverage CA: ComASP^®^ 89.5%; UMIC^®^ 92.4; DD 85.7%
Average EA: ComASP^®^ 78.7%; UMIC^®^ 82%ErrorsBias range: ComASP^®^ −36.1; UMIC^®^ −42.3–−11.4%

Average VME: ComASP^®^ 0%; UMIC^®^ 0%; DD 12%
Average ME: ComASP^®^ 1.2%; UMIC^®^ 1.9%; DD 6.4%
Average DD ATU: 14.5%
VMEs are prominent in DD within 17–22 mm.Test conditionsMedia and disk combinations strongly influence performance. 
Confirmatory BMD testing is critical for isolates in the 17–22 mm zone.

## 6. Expert Opinion and Discussion

FDC represents a crucial option for treating infections caused by MDR GN bacteria, particularly due to its unique mechanism of action as a siderophore cephalosporin. Its ability to bypass many resistance mechanisms, including efflux pumps and porin loss, makes it especially effective against strains producing metallo-β-lactamases, which are typically resistant to most available β-lactams. Additionally, FDC shows promising activity against *Acinetobacter* spp., a pathogen often associated with severe MDR infections, positioning it as a valuable therapeutic option in challenging clinical scenarios. For carbapenem-resistant *A. baumannii* isolates, however, the interpretation of susceptibility results requires caution, as we also report in this review, some discrepancies between MIC values and clinical outcomes have been observed [62]. These data highlight the importance of ongoing efforts to optimize AST methods and interpretive criteria for better decision-making.

Evaluating antimicrobial susceptibility to FDC involves unique challenges due to its dependency on low iron levels for efficacy, a mechanism designed to mimic the human body’s low iron availability [67,76]. Consequently, the BMD method requires using ID-CAMHB, which has been established as the reference standard. Nonetheless, preparing this medium proves cumbersome for the routine operations of clinical laboratories, highlighting the need for more adaptable and efficient methodologies. The variability in interpretive guidelines from standard-setting bodies like CLSI, FDA, and EUCAST further complicates susceptibility assessments for FDC. Moreover, wider MIC distributions observed in wild-type strains compared to other antibiotics might be attributable to variations in iron transporter and siderophore expression, which could modulate drug uptake, leading to MIC variability rather than indicating poor reproducibility of the BMD method [66]. The ATU serves as an important indicator within the testing process, alerting to potential interpretative challenges. As agreed by the expert panel, results within this zone can prompt actions such as (1) retesting, (2) applying alternative methodologies, or (3) reporting uncertainties in the findings.

Given these complexities, the Panel identified a need for developing guidance frameworks tailored to FDC AST, especially for pathogens like Enterobacterales, *P. aeruginosa*, and *A. baumannii*, the primary GN bacteria responsible for difficult-to-treat infections. These frameworks could be helpful in addressing specific challenges clinical microbiologists face in their daily routines and facilitating rapid and effective clinical downstream decision-making in treating infections caused by MDR-GN bacteria.

The panel indicates DD as a valuable initial screening method in FDC susceptibility testing for Enterobacterales and CRE. It typically achieves CA rates that exceed 88% and, in some cases, reach as high as 100% [67,70,71]. This level of reliability, coupled with the method’s low or null rates of VMEs, underscores its suitability for assessing FDC susceptibility, particularly in settings where rapid and reliable results are crucial [67,70,71]. As VMEs were relatively uncommon, the occurrence of MEs suggests that confirmation with BMD should be focused on resistant results, which are more prone to misclassification and carry greater clinical implications [67,70,71]. This practice is encouraged to ensure the accuracy of antimicrobial susceptibility testing, given the low rate of FDC resistance/reduced-susceptibility. However, it should be noted that the performance of DD can be significantly influenced by the choice of disks and media [60]. For example, studies have shown suboptimal results when using BioRad Mueller-Hinton agar combined with Liofilchem disks, particularly with a high incidence of VMEs among CRE isolates [60]. This finding points to the critical need for meticulous selection and standardization of materials in clinical laboratory settings to ensure test accuracy.

Among the MIC-based approaches, we agreed that the UMIC^®^ FDC system is reliable as an adjunct to DD, boasting EA rates above 90% and CA rates over 83%, although with VMEs noted in some isolates as high as 16.7% [52,68,70,72]. Its valid performances, which present only a slight tendency to undercall MICs, suggest that it could serve well in conjunction with DD when standard BMD is not feasible. Although the Sensititre™ panel performs best for the Enterobacterales group, particularly in terms of categorical agreement (CA), it is essential to note that the data come from a single study that evaluated this panel. Furthermore, at the time of writing, the Sensititre™ panel with cefiderocol had not been approved for in vitro diagnostic use in Europe.

The Panel agreed that this integration approach, in line with other authors’ results, could provide a more comprehensive assessment of FDC susceptibility and offset some of the feasibility limitations observed with conventional BMD techniques [67,70].

On the other hand, MTS has been found inadequate for this testing context due to significant accuracy and reliability issues [60,67,71]. Likewise, the ComASP^®^ cefiderocol microdilution panel has yielded limited and less satisfactory data than the UMIC^®^ FDC system [68,69].

Similarly, for *P. aeruginosa*, the UMIC^®^ FDC system emerges as the most accurate commercial BMD method currently available with CA and EA > 90% and no VME and low ME (2% and 8%) [70,72]. In contrast, the performance of MTS, which, despite being marketed explicitly for use with *P. aeruginosa*, tends to yield suboptimal results [73]. The reliance on DD as a standalone test is not suggested, as while it shows performances in terms of CA rates ranging from 78% to 100% and low ME rates, significant variations in inhibitory zones depending on the disk and MHA combinations, even among quality control strain [67,69,70,73]. Furthermore, the ATU as a sole indicator does not adequately address the interpretative challenges associated with this testing.

**Figure 4:**
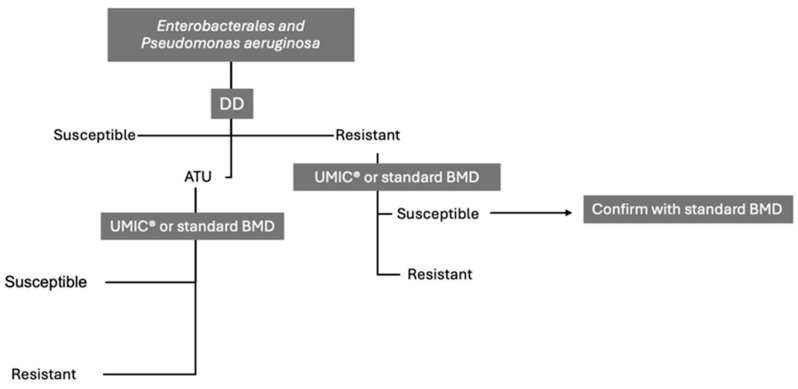
Decision Framework for Cefiderocol Susceptibility Testing in Enterobacterales and *P. aeruginosa* based on expert opinion informed by the literature review. Abbreviations: DD, disk diffusion; BMD, Broth Microdilution; ATU, Area of Technical Uncertainty.

Based on these considerations, the Panel proposed a decision-making framework for testing FDC susceptibility in Enterobacterales and *P. aeruginosa*, which initiates with DD to preliminarily screen isolates as susceptible, non-susceptible, or falling within the area of technical uncertainty (ATU) (Figure 4). For isolates deemed non-susceptible by DD, we suggest confirmatory testing using either the UMIC^®^ FDC system or, where available, the standard BMD technique with iron-depleted media. Also, if feasible, we suggest sending the sample to a reference laboratory. Following this, if UMIC^®^ is used and the result is susceptible, a standard BMD confirmation step is recommended. However, no further repetition is required if the initial confirmatory test has already been performed with standard BMD. For isolates within the ATU, we recommend retesting with UMIC^®^ or standard BMD to refine the categorization. DD results indicating susceptibility do not routinely require confirmation. This structured approach to testing is designed to enhance the reliability of the results by systematically addressing the interpretative challenges posed by FDC susceptibility testing, thereby utilizing the strengths of each method in sequence to ensure accurate and dependable outcomes (Figure 4).

Considering the available data for *A. baumannii* complex. DD is applicable as it shows acceptable performance in terms of CA but is conditional on disk types and media, with maximum values reaching >90% [58,59,67,69,70,75]. In this setting, there is a critical need to reevaluate the sensitivity cut-offs because numerous strains, showing inhibition zone diameters ranging between 17 mm and 22 mm, which would categorize them as susceptible, prove to be non-susceptible when tested using the reference BMD method. Despite commercial BMD methods not fully meeting ISO standards, they do exhibit valuable CA rates of 88–95.9% and 89–97.4% for the ComASP^®^ cefiderocol microdilution panel and UMIC^®^ FDC system, respectively [59,70,72,73]. MTS is not recommended in this context due to its limited accuracy [59].

**Figure 5:**
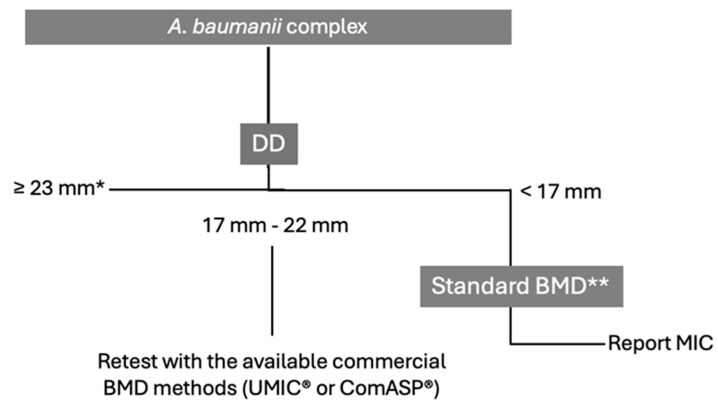
Decision Framework for Cefiderocol Susceptibility Testing in *A. baumanii* complex based on expert opinion informed by the literature review. Abbreviations: DD, disk diffusion; BMD, Broth Microdilution. * could be reported in a note as a MIC ≤ 2; ** very preliminary information: possible change from 18 (ECOFFS) to 19 (breakpoint).

For *A. baumannii* complex, the Panel agreed on a decision-making framework that begins with disk diffusion (DD). We recommend the following stepwise approach: isolates with inhibition zones < 17 mm are to be considered non-susceptible and should undergo confirmatory testing using a standard BMD method with iron-depleted media; those with zones between 17 and 22 mm should be retested using a commercial BMD method such as UMIC^®^ or ComASP^®^. Importantly, we acknowledge that this approach may not eliminate the risk of VMEs, particularly in isolates near the current ECOFF, and we explicitly recommend further confirmation with BMD whenever possible, especially in critical clinical contexts or when inhibition zones fall within this intermediate range. Isolates with zones ≥ 23 mm may be considered susceptible without further testing (Figure 5). Although our proposed framework for *A. baumannii* susceptibility testing appears less complex than other pathogens, this reflects the currently limited availability of harmonized approaches and standardized breakpoints for this species.

The first note specifies that for inhibition zones equal to or greater than 17 mm, a footnote should be appended to the report indicating an expected MIC value of ≤2 mg/L. The second note addresses potential forthcoming adjustments in the reference values, suggesting a shift from the current epidemiological cut-off (ECOFF) of 18 to a new breakpoint of 19. These amendments aim to refine the interpretative accuracy of test results and ensure that susceptibility reporting remains aligned with evolving scientific standards.

Our proposed decision-making frameworks aim to strike a pragmatic balance between diagnostic accuracy and practical feasibility. It is oriented toward reducing the likelihood of overestimating resistance, which could lead to unnecessary therapeutic exclusions. Moreover, while more conservative strategies—focused on minimizing the risk of false susceptibility—may offer advantages in selected contexts, they typically require broader and repeated implementation of reference BMD testing, which is not always operationally sustainable. That said, alternative confirmation by standard BMD or reference laboratory referral remains essential where UMIC^®^ is not yet available. In these terms, recent multicenter data from Koeth et al. suggest that ComASP^®^ may offer reliable performance for *A. baumannii*, supporting its use as an alternative to UMIC^®^ for confirmation testing in settings where access is limited [77].

## 7. Conclusions

To date, real-world evidence has highlighted the role of FDC in treating infections caused by carbapenem-resistant or extensively resistant GN bacteria, particularly in critically ill or immunocompromised patients. Accurate and efficient testing is crucial to ensure that this antimicrobial agent can be utilized effectively in clinical scenarios, where it represents an essential treatment option.

Based on the evidence reviewed and expert consensus, we propose a stepwise testing framework that prioritizes clarity, feasibility, and clinical relevance. Disk diffusion may be used as an initial screening method but isolates with inhibition zones in the ATU range or with reduced susceptibility should be confirmed using commercial BMD-based methods, preferably UMIC^®^ or ComASP^®^.

A limitation of our study is the relatively small number of studies currently available for review, positioning our work as a snapshot of the present state of AST for FDC. Recognizing this, we briefly identified the current research gaps and suggested future directions for investigation, as outlined in Box 4. While the identified gaps reflect a broad spectrum of challenges, we acknowledge that not all can be addressed in a single research initiative. As a strategic focus, we suggest prioritizing the standardization of AST methodologies and the inclusion of diverse clinical isolates in validation studies. These steps would provide an essential foundation upon which further, more granular investigations can be built over time. This approach underscores the commitment to advancing the field and supporting the clinical use of cefiderocol as an indispensable option in the antibiotic arsenal.

Box 4 **Gaps in the current research and suggestions for future studies.**

**Specific Gaps**

**Implications**

**Future Research Suggestions**
Heterogeneity in Study DesignDiverse methodologies across studies lead to difficulty in comparing and synthesizing results.Standardize and report frameworks in AST studies to ensure consistency and comparability.Small Sample SizesLimited sample sizes reduce the statistical power and generalizability of findings.Implement multicenter studies to gather larger, more diverse bacterial isolates and enhance the representativeness of results.Use of Standard Reference Strains OnlyReliance solely on ATCC strains might not accurately represent clinical scenarios.Incorporate clinical isolates with known resistance profiles as controls in testing protocols.Longitudinal SurveillanceEvolving resistance patterns are not adequately monitored over time, affecting the relevance of AST protocols.Conduct longitudinal surveillance studies to monitor resistance evolution and method efficacy.Variations in ATUChanges in ATU boundaries may impact susceptibility interpretations, increasing variability in results.Evaluate the influence of ATU variations on susceptibility testing outcomes and explore strategies to minimize inconsistencies.

## Figures and Tables

**Table 1:** CLSI, EUCAST, and FDA cefiderocol breakpoints.

	MIC Breakpoint (mg/L)			Disk Zone Diameter Breakpoint (mm)			
Organism	CLSI	EUCAST	FDA	CLSI	EUCAST	ATU (EUCAST)	FDA
Enterobacterales	≤4 (S), 8 (I), ≥16 (R)	≤2 (S), >2 (R)	M-100 standard is recognized	≥16 (S), 9–15 (I), ≤8 (R)	≥23 (S), <23 (R)	21–23	M-100 standard is recognized ^§^
*Pseudomonas aeruginosa*	≤4 (S), 8 (I), ≥16 (R)	≤2 (S), >2 (R)	≤1 (S), 2 (I), ≥4 (R)	≥18 (S), 13–17 (I), ≤12 (R)	≥22 (S), <22 (R)	20–21	≥22 (S), 13–21 (I), ≤12 (R)
*Acinetobacter baumannii* complex	≤4 (S), 8 (I), ≥16 (R)	IE	≤1 (S), 2 (I), ≥4 (R)	≥15 (S) **	Note *		≥19 (S), 12–18 (I), ≤11 (R)
*Stenotrophomonas maltophilia*	≤4 (S), 8 (I), ≥16 (R)	IE	Not specified	≥15 (S)	Note °		≥17 (S)

* Zone diameters of ≥17 mm for the cefiderocol 30 µg disk correspond to MIC values below the PK-PD breakpoint of S ≤ 2 mg/L. IE: Insufficient evidence exists that the organism is a good target for therapy with the agent. ATU, Area of technical Uncertainty. ** DD ≤ 14 mm should not be interpreted or reported because it occurs with resistant, intermediate, and susceptible isolates. For these, do not report without performing a MIC test. ° Zone diameters of ≥20 mm for the cefiderocol 30 µg disk correspond to MIC values below the PK-PD breakpoint of S ≤ 2 mg/L. ^§^ Clinical efficacy was shown for Escherichia coli, Klebsiella pneumoniae, Proteus mirabilis, and Enterobacter cloacae complex in patients with complicated urinary tract infections (cUTI) and for *Escherichia coli*, *Klebsiella pneumoniae*, *Enterobacter cloacae* complex, and *Serratia marcescens* in patients with hospital-acquired bacterial pneumonia and ventilator-associated bacterial pneumonia (HABP/VABP).

## Data Availability

No new data were created or analyzed in this study.

## Supplementary Materials

The following supporting information can be downloaded at: https://www.mdpi.com/article/10.3390/antibiotics14080760/s1, Table S1: Comparative Analysis of AST Methods for Enterobacterales; Table S2: Comparative Analysis of AST Methods for *Pseudomonas aeruginosa*; Table S3: Comparative Analysis of AST Methods for *A. baumannii complex.*

## Abbreviations

The following abbreviations are used in this manuscript:

FDC	Cefiderocol
AST	Antimicrobial susceptibility testing
BMD	Broth microdilution
ID-CAMHB	Iron-depleted cation-adjusted Mueller-Hinton broth
ATUs	Areas of technical uncertainty
CRE	Carbapenem-resistant Enterobacterales
DD	Disk diffusion
S	Susceptible
R	Resistant
AMR	Antimicrobial resistance
GN	Gram-negative
ICU	Intensive Care Units
CAESAR	Central Asian and European Surveillance of Antimicrobial Resistance
EARS-Net	European Antimicrobial Resistance Surveillance Network
MBLs	metallo- β -lactamases
CR	carbapenemase-resistant
CA	Categorical Agreement
EA	Essential Agreement
VME	Very Major Errors
ME	Major Errors
mE	minor errors
NS-FDC	non-susceptibility to FDC
MH	Mueller-Hinton
iROMPs	iron-regulated outer membrane proteins
ECOFF	epidemiological cut-off
