# Supplementary material for: Evaluating Antimicrobial Susceptibility Testing Methods for Cefiderocol: A Review and Expert Opinion on Current Practices and Future Directions
Source: Antibiotics (Basel). 2025 Jul 28;14(8):760. doi: 10.3390/antibiotics14080760 (PMC12382653; doi:10.3390/antibiotics14080760)
Supplement: Supplementary file 1 [file antibiotics-14-00760-s001.zip › antibiotics-3728622-supplementary.pdf]

SUPPLEMENTARY MATERIALS

Table S1. Comparative Analysis of AST Methods for Enterobacterales.

Type of ID-CAMHB	Disk Diffusion				ComASP®					UMIC®					Sensititre™				
	ATU%	CA%	VME%	ME%	CA%	EA%	Bias%	VME%	ME%	CA%	EA%	Bias%	VME%	ME%	CA%	EA%	Bias%	VME%	ME%
Collection characteristics	Aggregated data on strain collection not available																		
Frozen panels																			
**Dortet 2023 (n=180) [72]**										87.8	91.7	-25.0	2.7	0.5					
Collection characteristics	Beta-lactamases producers (3), KPCs (34), NDM producers (22), VIM producers (10), OXA producers (7), CTX-M producers (11), SHV and TEM producers (3)																		
Bruker Daltonics	Oxoid, Liofilchem, and Mast Diagnostics on MHA																		
**Bianco 2024 (n=90) [70]**	37.8	98.2 (Mast group) - 100 (others)	0	0 (others) - 1 (Mast group)						81.1	91.1	+26.7	1.1	7.7					
Collection characteristics	44% CRE i.e. KPC (21), NDM and OXA-181 (3), OXA-181 (1), and (SME) (1)																		
Sensititre™ (Thermo Fisher)	Mast Diagnostics disks and HardyDisks on MHA (Becton Dickinson)																		
**Morris 2021 (n=58)* [67]**	NA	88 (CLSI)-90 Mast<br>88-91(CLSI)-Hardy	0-0 Mast<br>0-0 Hardy	NA(CLSI)-14 Mast<br>0 (CLSI)-17 Hardy															
Collection characteristics	KPC producers (114), ceftazidime/avibactam resistant (44), NDM producers (19), VIM-producers (31), OXA-48-like producers and KPC/VIM co-producers (10)																		
ID-CAMHB BMD	Oxoid disks and CA-MH agar (Becton-Dickinson)																		
**Bianco 2023 (n=178) [69]**	21.4				92.1	NA	NA	2.6	5.3										
Collection characteristics	Carbapenemase of Ambler's class A, B, D, multiple carbapenemases and non carbapenemase-producers																		

Frozen panels	Liofilchem disks on MHA (Biorad, Marnes la Coquette, France)																		
**Bonnin 2022 (n=100) [60]**	21.3	77	35.9	1.6											95	87	NA	2.8	1.6
Collection characteristics	CRE (21 OXA-48 like, 15 NDM, 4 VIM, 2 KPC, 2 IMP, 1 FRI-1, 1 GIM-1, 1 GES-5, 1 NmcA, 1 OXA-372, 4 double-producing OXA-48 like and NDM, 1 NDM + KPC and 1 NDM + VIM) and 5 strains with high-level expression of EBSL or AmpC associated with decreased permeability of the outer membrane																		
Sensititre™ (Thermo Fisher)																			
**Emeraud 2023 (n=60) [68]**					83.3	76.7	-6.7	34.5 (17.2 out of EA)	0	83	91.7	-10	24.1 (6.9 out of EA)	0					
Collection characteristics	KPC-producing *K. pneumoniae*, 42.6% resistant to CAZ-AVI and/or MER-VAB and/or IMIREL. 46.9% of the CAZ-AVI R strains carried blaKPC3, 50% mutated blaKPC3 and 3.1% blaKPC2																		
ID-CAMHB BMD	Liofilchem disks on regular non-supplemented MHA (Liofilchem)																		
**Bovo 2023 (n=75) [71]**	NA	92 (98.6**)	0	16.7 (2.8 **)															
Collection characteristics	NDM- *K. pneumoniae* isolates from bloodstream infections with MICs ranging between 0.5-2 µg/mL																		
ID-CAMHB BMD	Liofilchem disks on MHA plates (Biolife)																		
**Leonildi Poster 1641 ESCMID 2024 (n=30) [52]**	74	NA	NA	NA	NA	100	NA	NA	NA	NA	100	NA	NA	NA					

Data are referred to EUCAST breakpoints where not otherwise indicated. BMD, brothmicrodilution; DD, disk diffusion; EA, Essential Agreement; CA, Categorical Agreement; VME, Very major error; ME, Major error; ATU, Areas of Technical Uncertainty; NA, not available; ID-CAMHB, Iron-Depleted Cation-Adjusted Mueller-Hinton Broth. *minor error (mE)% 12 (CLSI) Mast and 9 (CLSI) Hardy; **Excluding the discordant strains within the ATU.

Table S2. Comparative Analysis of AST Methods for Pseudomonas aeruginosa.

**Type of BMD**	**Disk Diffusion**				**ComASP®**					**UMIC®**					**Sensititre™**				
**ID- CAMHB**	**ATU%**	**CA%**	**VME%**	**ME%**	**CA%**	**EA%**	**Bias%**	**VME%**	**ME%**	**CA%**	**EA%**	**Bias%**	**VME%**	**ME%**	**CA%**	**EA%**	**Bias%**	**VME%**	**ME%**
Collection characteristics	Carbapenem-resistant *P. aeruginosa*																		
Sensititre™ (ThermoFisher)	Mast Diagnostics disks and HardyDisks on MHA (Becton Dickinson)																		
**Morris 2021 (n=14)* [67]**	NA	86-86 (CLSI) Mast<br>79-93(CLSI) Hardy	0 (CLSI) – 25 Mast<br>0 (CLSI) – 25 Hardy	0 (CLSI) – 10 Mast<br>0 (CLSI) – 14 Hardy															
Collection characteristics	Carbapenem-resistant *P. aeruginosa. 10 harbored blaVIM*																		
**ID-CAMHB BMD**	Oxoid disks and CA-MH agar (Becton-Dickinson)																		
**Bianco et al. 2023 (n=42) [69]**	11.9	NA	NA	NA	100	NA	NA	0	0										
		Aggregated data on strain collection not available																	
Frozen panels																			
**Dortet 2023 (n=49) [72]**										98	93.9	+12.2	0	2.04					
Collection characteristics	13 strains no carbapenemase, 6 strains VIM-related carbapenemase, 5 strains IMP-related carbapenemase, 7 strains KPC or TEM-related carbapenemase, 5 strains NDM-related carbapenemase, the other mixed OXA-related carbapenemase																		
Bruker Daltonics	Oxoid, Liofilchem, and Mast Diagnostics on MHA																		
**Bianco 2024 (n=21) [70]**	40	95.8-100	2.5	0						90	92.5	<30	0	8					
Collection characteristics	40 producing ESBL, 52 producing carbapenemases (including 11 VIM-2), and 15 producing both types of enzymes.																		
ID-CAMHB BMD	Mast Diagnostic, Liofilchem, and Oxoid disks on MHA plates from 6 manufacturers**																		
**Devoos 2023 (n=150) [73]**	NA	78-89.3	18.7-84.3	low°											86.7	69.3	-9.3 - +77.5	2.7	8

		(overall 84)																	

Data are referred to EUCAST breakpoints where not otherwise indicated. BMD, brothmicrodilution; DD, disk diffusion; EA, Essential Agreement; CA, Categorical Agreement; VME, Very major error; ME, Major error; ATU, Areas of Technical Uncertainty; NA, not available; ID-CAMHB, Iron-Depleted Cation-Adjusted Mueller-Hinton Broth. *minor error (mE)% 14(CLSI) Mast and 7 (CLSI) Hardy; ** Oxoid, Thermofisher scientific; Mast Diagnostic, Amiens; Becton Dickinson; I2a diagnostics,Montpellier, France; Bio-Rad, Marnes-la-Coquette, France and bioM erieux, Marcy-L'Etoile,France); °the exact value was not reported.

Table S3. Comparative Analysis of AST Methods for *A. baumannii complex*

Type of BMD	Disk Diffusion				ComASP®					UMIC®				
ID- CAMHB	ATU %	CA %	VME %	ME %	CA%	EA%	Bias%	VME%	ME%	CA%	EA%	Bias%	VME%	ME%
Collection characteristics	Carbapenem-resistant *A. baumannii* complex													
Sensititre™ (ThermoFisher)	MASTDISCS (Mast Group) disks and HardyDisks (Hardy Diagnostics, Santa Maria, CA) on MH agar (Becton Dickinson													
**Morris 2021*<br>(n=14) [67]**	NA	71-86 (CLSI) Mast<br>64-61 (CLSI) Hardy	0 (CLSI) – 11 Mast<br>0 (CLSI) – 33 Hardy	0 (CLSI) – 20 Mast<br>25 (CLSI) – 20 Hardy										
Collection characteristics	Carbapenem-resistant A. baumannii. 46 OXA-23 carbapenemase and 2 OXA-23/NDM co-producers													
ID-CAMHB BMD	Oxoid disks and CA-MH agar (Becton-Dickinson)													
**Bianco 2023<br>(n=48) [69]**	14.5	97.9	0	2.1	85.7	NA	NA	0	0					
Collection characteristics	Aggregated data on strain collection not available													
Frozen panels														
**Dortet 2023<br>(n=44) [72]**										90.9	84.1	-11.4	6.81	2.27
Collection characteristics	29/39 blaOXA-23-positive alone or in combination with other mechanism, other (i.e. NDM-1; OXA-66, ADC-30, OXA-58)													
Bruker Daltonics	Oxoid, Liofilchem, and Mast Diagnostics on MHA													
**Bianco 2024<br>(n=39) [70]**	NA	94.9	3	3						97.4	89.7	NA	NA	NA
Collection characteristics	Plasmid-borne carbapenemases in 94/100 isolates. OXA-23 (63%), OXA-72 (14%), OXA-58 (3%), and OXA-40 (1%)													
ID-CAMHB BMD	Liofilchem disk on ID-CAMH, Biomerieux and Liofilchem CAMH-agar plates													
**Kolesnik-Goldmann 2023<br>(n=100) [59]**	NA	86 ID-CAMH, 87 Biomerieux, 84 Liofilchem	7 ID-CAMH, 11 Biomerieux, 16 Liofilchem	7 ID-CAMH, 2 Biomerieux, 0 Liofilchem	88 -86 (CLSI)	76- 76 (CLSI)	NA	7-6 (CLSI)	5-0 (CLSI)	89 – 86 (CLSI)	76- 76 (CLSI)	NA	9-3 (CLSI)	2-1 (CLSI)

		95.1 ID-CAMH (CLSI), 91.1 Biomerieux (CLSI), 89.2 Liofilchem (CLSI)	1.2 ID-CAMH (CLSI), 11 Biomerieux (CLSI), 6.4 Liofilchem (CLSI)	3.7 ID-CAMH (CLSI), 4.4 Biomerieux (CLSI), 4.3 Liofilchem (CLSI)										
Collection characteristics	70 carbapenemase producers (OXA-type 58 strains, NDM-type in 11 strains, IMP-type enzyme in 1strain), 22 with two carbapenemase-encoding genes. ESBL in 17 strains. 1 strain GES-11 ESBL													
ID-CAMHB BMD		Oxoid, Liofilchem and MAST disks on MH agar (Becton-Dickinson)												
**Jeannot 2023 (n=97) [58]**	NA	72.2 (Oxoid)-78.4 (Liofilchem) 81.4 (MAST)	42.9 (Oxoid)- 50 (Liofilchem) 64.3 (MAST)	0	95.9	81.4	- 36.1	NA	NA	93.8	78.4	-42.3	NA	NA
Collection characteristics	104 carbapenem-susceptible *A. baumannii* complex and 364 carbapenem-resistant *A. baumannii* complex													
ID-CAMHB BMD	Mast disks and MHA Oxoid													
**Liu et al, 2023 (n=468) [75]**	NA	98.1 (CLSI) - 97.0	0.9 (CLSI) -1.9	NA										

Data are referred to EUCAST breakpoints where not otherwise indicated. BMD, brothmicrodilution; DD, disk diffusion; EA, Essential Agreement; CA, Categorical Agreement; VME, Very major error; ME, Major error; ATU, Areas of Technical Uncertainty; NA, not available; ID-CAMHB, Iron-Depleted Cation-Adjusted Mueller-Hinton Broth; ID-CAMH, Iron-Depleted Cation-Adjusted Mueller-Hinton.. *minor error (mE)% 28CLSI) Mast and 14 (CLSI) Hardy.
